# Results from a Meta-analysis of Combination of PD-1/PD-L1 and CTLA-4 Inhibitors in Malignant Cancer Patients: Does PD-L1 Matter?

**DOI:** 10.3389/fphar.2021.572845

**Published:** 2021-02-25

**Authors:** Yuqian Feng, Huimin Jin, Kaibo Guo, Yuying Xiang, Yiting Zhang, Wurong Du, Minhe Shen, Shanming Ruan

**Affiliations:** ^1^ The First Clinical Medical College of Zhejiang Chinese Medical University, Hangzhou, China; ^2^ Department of Medical Oncology, The First Affiliated Hospital of Zhejiang Chinese Medical University, Hangzhou, China

**Keywords:** combination immunotherapy, PD-L1, PD-1, CTLA-4, efficacy

## Abstract

**Background:** Combination therapy with immune checkpoint inhibitors (ICIs) has been widely used for clinical treatment in recent years, which has a better survival benefit. However, not all patients can derive clinical benefit from combination immunotherapy. Therefore, it is necessary to explore the biomarkers of combination immunotherapy.

**Methods:** We retrieved articles from electronic databases including PubMed, EMBASE and Cochrane. The statistical analysis was performed using RevMan software. Progression free survival (PFS), overall survival (OS) and objective response rate (ORR) were the outcome indicators. In the unselect population, we compared combination therapy with other treatments. In addition, we also conducted subgroup analysis on PFS, OS and ORR according to PD-L1 status.

**Results:** Seven studies were included in the analysis for a total of 3,515 cases. In the unselected population, we found that combination therapy has longer PFS, OS, and better ORR than other treatments for cancer patients. The longer PFS was showed in PD-L1 ≥ 5% cases (HR = 0.64, 95% CI: 0.56–0.76; *p* < 0.001) than PD-L1 ≥ 1% cases (HR = 0.72, 95% CI: 0.66–0.79; *p* < 0.001), while ORR and OS have not related to the status of PD-L1.

**Conclusion:** This study supported the efficacy of combination therapy with immune checkpoint inhibitors (ICIs), and also showed that PFS in patients with malignant tumors is positively correlated with PD-L1 expression. Due to the limited number of trials included, more high-quality clinical randomized controlled trials should be conducted to confirm the review findings.

## Introduction

Due to the limited therapeutic effect, drug resistance, and adverse events of chemotherapy in malignant tumors (Islam et al., 2019), many new anti-tumor methods have emerged, such as traditional Chinese medicine, molecular-targeted therapy and immunotherapy (Ishihara et al., 2021; Kong et al., 2020; Tang et al., 2020). In particular, immune checkpoint inhibitors (ICIs) has become a hot topic in recent years (Darvin et al., 2018). As expected, ICIs have provided a surprising breakthrough in the treatment of cancer. It has shown a more durable response and longer survival in a variety of cancers (Borghaei et al., 2015; Larkin et al., 2015; Cheng et al., 2018). Present studies have confirmed that the cancers harboring highly immunogenic mutations are sensitive to ICIs, such as melanoma, RCC, and NSCLC (Topalian et al., 2012).

Ipilimumab and tremelimumab, which both target CTLA-4, can prevent normal down-regulation of T cells and prolong T-cell action (Tarhini and Kirkwood 2008; Darvin et al., 2018). Durvalumab is a selective and high-affinity human immunoglobulin G1 monoclonal antibody, which blocks PD-L1 binding to PD-1 and CD80 (Stewart et al., 2015). While nivolumab is a human monoclonal antibody that selectively blocks the PD-1 receptor on the surface of cytotoxic T cells to prevent downregulation of the immune response in malignant tumor cells induced by PD-L1 (Minguet et al., 2016).

Immunotherapy combined with chemotherapy or targeting drugs has shown capability to extend patient survival time in multiple cancers (Robert et al., 2011; Reck et al., 2016; Pal et al., 2020). ICIs plays a therapeutic role by activating T cells in the tumor immune microenvironment by suppressing immune checkpoints. However, T cells activated by anti-PD-1/PD-L1 or anti-CTLA-4 may be inhibited by other immunosuppressive cells or factors in the tumor immune microenvironment (Jia et al., 2020). Hence, clinical trials for dual immunotherapy are also emerging. The combination of nivolumab and ipilimumab reported a longer survival time and progression-free survival than either nivolumab or ipilimumab (Larkin et al., 2015). Similarly, the combination of durvalumab and tremelimumab was also more effective than either of them (Planchard et al., 2020). This may be related to the dual inhibitory effects of PD-1/PD-L1 and CTLA-4 pathways, which enhance the anti-tumor efficacy (Curran et al., 2010).

Expression of PD-L1 is a potential prognostic biomarker for cancer patients undergoing PD-1/PD-L1 targeting therapy (Darvin et al., 2018). A previous meta-analysis contained about 6,000 patients with different cancers, has suggested that PD-L1 expression is significantly associated with clinical response to anti-PD-1/PD-L1 in patients with non-squamous NSCLC and melanoma (Gandini et al., 2016). However, few meta-analyses have been conducted on the relationship between the efficacy of combination immunotherapy and the expression of PD-L1. Whether combination immunotherapy can increase the clinical efficacy compared with other treatments, and whether its efficacy is related to the expression of PD-L1? Therefore, we reviewed the relevant clinical trials and performed this meta-analysis.

## Materials and Methods

This systematic review and meta-analysis followed the Preferred Reporting Items for Systematic Reviews and Meta-Analyses (PRISMA) statement (Moher et al., 2009). The protocol for this systematic review was registered on the PROSPERO International prospective register of systematic reviews (CRD42020182767) and is available in full on the website at http://www.crd.york.ac.uk/PROSPERO.

### Search Strategy

Two investigators (Y.Q.F. and H.M.J.) independently searched PubMed, EMBASE and Cochrane Library databases for eligible studies from inception to March 31, 2020. The search terms include “Programmed Cell Death 1 Receptor,” “Programmed death ligand 1,” “PD-1,” “PD-L1,” “CTLA-4 Antigen,” and “randomized controlled trial” (for details see Supplementary Material 1). We also manually reviewed the relevant literatures cited in the references to find additional eligible clinical trials. When different publications derived from the same trails, we only chose data from the most recent or appropriate report.

### Eligibility Criteria

The inclusion of the article was performed independently by two of investigators (Y.Q.F. and H.M.J.), and a third investigators (K.B.G.) was consulted in case of disagreement. The studies we included met the following criteria: a) in malignant cancer patients; b) anti-PD-1/anti-PD-L1 plus anti-CTLA-4 therapy is the treatment arm; c) control arm can be anything other than combination immunotherapy; d) Studies have data available for PD-L1 expressed related hazard ratio (HR) and 95% confidential interval (CI) of OS/PFS, or the number of patients with objective response in both the experimental group and the control group; e) randomized controlled trial; f) Each group has a sample size of more than 10 patients. Meanwhile, the exclusion criteria were as follows: a) not in malignant cancer patients; b) anti-PD-1/anti-PD-L1 plus anti-CTLA-4 therapy is not the treatment arm; c) combination immunotherapy is the treatment arm; d) Studies do not have data available for PD-L1 expressed related hazard ratio (HR) and 95% confidential interval (CI) of OS and PFS, the number of patients with objective response in both the experimental group and the control group; e) non-randomized controlled trial; animal studies; f) One of group has a sample size of less than 10 patients; g) only the abstract part, no full text.

### Data Extraction

The relevant data was extracted by two investigators (Y.Q.F. and H.M.J.) independently via a predefined data extraction form. Any disagreements were resolved through discussion to reach a final consensus, such as the inconsistency of the extracted data and the controversy over the inclusion of specific information. Study characteristics and outcome data were extracted from the included trials. From each trial, we extracted specific information on study number, the phase of study, first author name, publish year, treat line, cancer type, primary endpoint, study design, efficacy data and PD-L1 detection method.

### Bias Assessment

The risk of bias assessment was conducted by two reviewers (Y.Q.F. and H.M.J.) independently in accordance with the Cochrane Handbook for Systematic Reviews of Interventions (Version 5.1.0) (Higgins et al., 2011). For inconsistent opinions, the two reviewers resolved differences through discussion to achieve an agreement.

### Statistical Analysis

All the analyses were accomplished by RevMan software (Version 5.3 for Windows). Data from different trails were pooled via Mantel-Haenszel method with either fixed-effects model or random-effects model, depending on the degree of heterogeneity (statistically rather than clinically). Statistical heterogeneity was assessed with the Q-test and the I^2^ statistic. When *p* > 0.1 and I^2^ < 50%, the fixed-effects model was used; otherwise, the random-effects model was used. Time-to-event variables, including OS, PFS, HRs with 95% confidence intervals (CIs) were calculated for each study. For the dichotomous variables, risk ratios (RRs) with 95% CIs were calculated. A value of *p* < 0.05 was regarded to be statistically significant, and all tests were two sided.

## Results

### Search Results and Studied Characteristics

**FIGURE 1:**
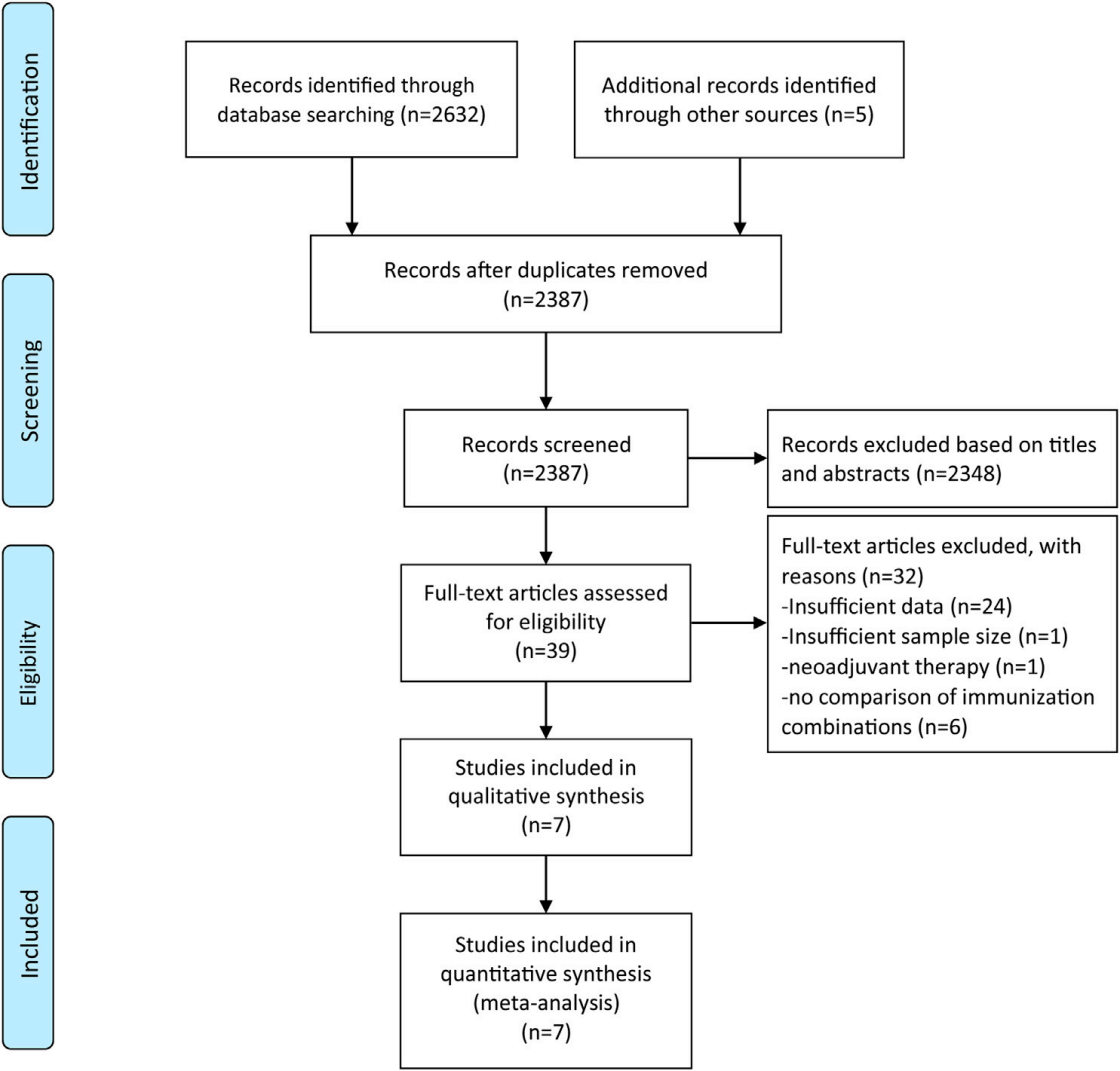
PRISMA chart.

A total of 2,637 articles were retrieved from three electronic databases using the comprehensive search strategy. Duplicate articles were eliminated through automatic and manual re-check, leaving 2,387 articles. We then browsed through the titles and abstracts to weed out 2,348 completely unrelated articles. After the title and abstract screening, 39 records were considered for full-text evaluation, of which seven records were included in the final analysis (Hodi et al., 2016; Janjigian et al., 2018; Motzer et al., 2018; Hellmann et al., 2019; Larkin et al., 2019; Planchard et al., 2020; Rizvi et al., 2020). Each of step was performed and proofread by two investigators independently. The study inclusion procedure is shown in Figure 1.

All included studies were published between 2016 and 2020. Among them, five trials were first-line treatments and two trials were third-line treatments or later. A total of 4,414 patients from 7 RCTs were enrolled in our present meta-analysis, including 1,928 in treatment arm and 2,486 in the control arm. The treatment regimen of the experimental group was nivolumab plus ipilimumab or durvalumab plus tremelimumab (Table 1). The survival data of the overall population and the PD-L1 positive population are shown in Supplementary Table S1.

**TABLE 1:** Characteristics of included studies.

Study (phase)	First author	Year	Treat line	Cancer type	Primary endpoint	Treatment arm (No. of patients)	Control arm (No. of patients)	Experimental drug/control arm	PD-L1 detection
NCT01844505 (phase 3)	James Larkin	2019	First line	Melanoma	ORR, OS, PFS	314	316/315	N+I/I/N	NR
NCT01927419 (phase 2)	F Stephen Hodi	2016	First line	Melanoma	ORR, OS, PFS	95	47	N+I/I	Bristol-Myers Squibb and Dako
NCT01928394 (phase 1/2)	Yelena Y. Janjigian	2018	≥Third line	Esophagogastric Cancer	ORR, OS, PFS	49	59	N+I/N	Dako North America, Carpinteria, CA
NCT02231749 (phase 3)	Robert J Motzer	2018	First line	Renal-Cell Carcinoma	ORR, OS, PFS	550	546	N+I/S	Dako PD-L1 IHC 28-8 pharmDx
NCT02352948 (phase 3)	D.Planchard	2020	≥Third line	Non–Small-Cell Lung Cancer	ORR, OS, PFS	174	118/117/60	D+T/Soc/D/T	VENTANA PD-L1 (SP263)
NCT02453282 (phase 3)	Naiyer A. Rizvi	2020	First line	Non–Small-Cell Lung Cancer	ORR, OS, PFS	163	162/163	D+T/C/D	NR
NCT02477826 (phase 3)	Matthew D. Hellmann	2019	First line	Non–Small-Cell Lung Cancer	ORR, OS, PFS	583	583	N+I/C	Agilent Dako

N, nivolumab; I, ipilimumab; D, durvalumab; T, tremelimumab; S, sunitinib; Soc, standard of care; C, chemotherapy; NR, not reported.

### Quality Assessment

**FIGURE 2:**
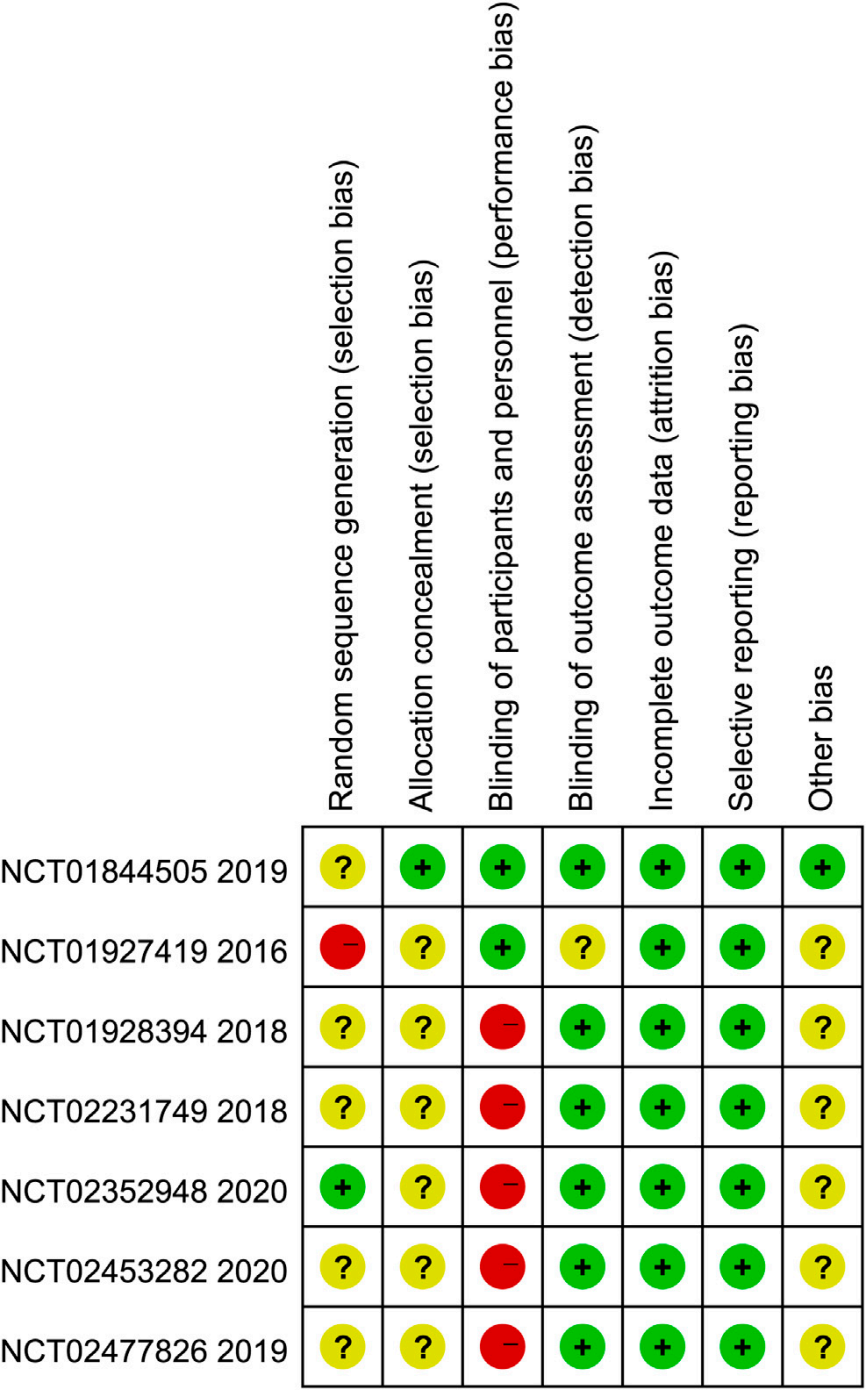
Risk of bias of included studies.

Figure 2 summarized the results of the quality assessment of seven eligible studies. In general, the included studies were judged to have a low risk of bias. Among them, one trial (Hodi et al.) had high risk of random sequence generation and five trials (Larkin et al.; Yelena et al.; Motzer et al.; Rizvi et al.; Hellmann et al.) were evaluated as unclear risk of bias. Only one trial (Larkin et al.) clearly reported the selection bias. We rated five trials (Yelena et al.; Motzer et al.; Planchard et al.; Rizvi et al.; Hellmann et al.) as high risk of performance bias, since they take different amounts of medication and no placebo was used. One trial (Hodi et al.) reported unclearly about blinding of outcome assessment.

### Efficacy

#### Progression Free Survival

**FIGURE 3:**
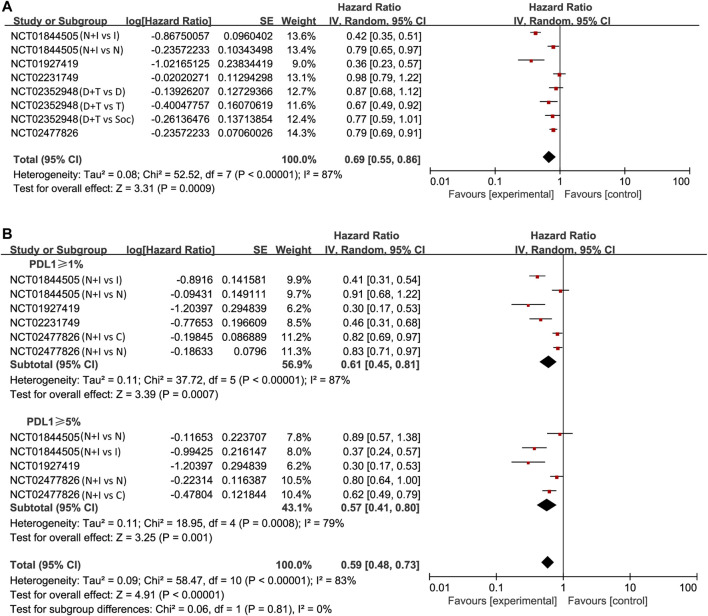
Forest plots of hazard ratios (HRs) for progression-free survival (PFS) comparing combination of immune checkpoint inhibitors with other treatments. **(A)** Unselected patients. **(B)** PD-L1 positive patients.

The pooled analysis in unselected cases showed improved PFS in the experimental arm (HR = 0.69, 95% CI: 0.55–0.86; *p* < 0.001, Figure 3A). The analysis was performed using a random-effects model (I^2^ = 87%). We then compared PFS in patients whose PD-L1 expression was ≥1 and ≥5%. The pooled analysis in PD-L1 ≥ 1% cases showed improved PFS in the experimental arm (HR = 0.61, 95% CI: 0.45–0.81; *p* < 0.001, Figure 3B) and even greater PFS improvement in PD-L1 ≥ 5% cases (HR = 0.57, 95% CI: 0.41–0.80; *p* = 0.001, Figure 3B).

#### Overall Survival

**FIGURE 4:**
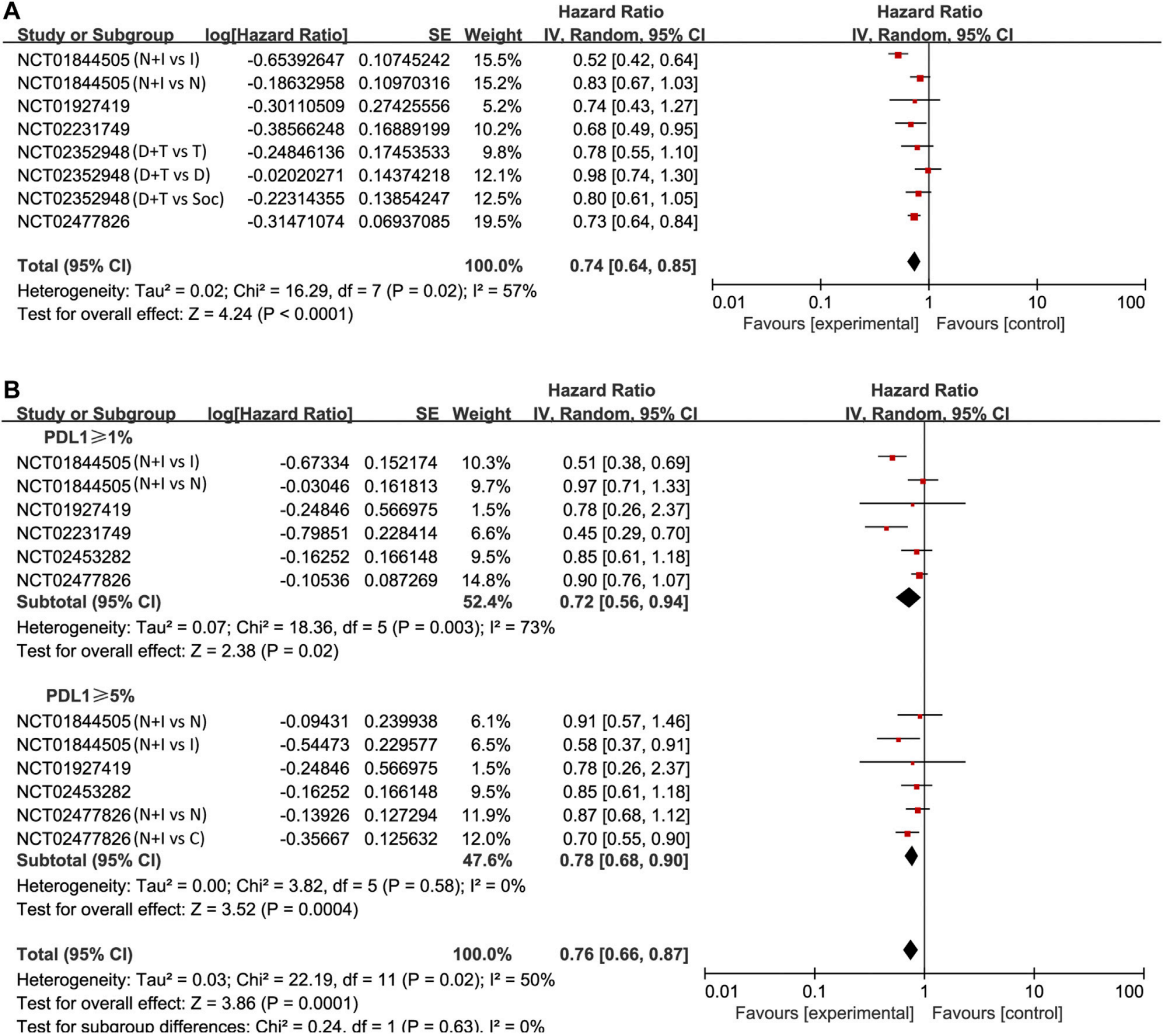
Forest plots of hazard ratios (HRs) for overall survival (OS) comparing combination of immune checkpoint inhibitors with other treatments. **(A)** Unselected patients. **(B)** PD-L1 positive patients.

We also found that PD-1/PD-L1 combined with CTLA-4 had a better effect than other treatments through the OS study of unselected cases (HR = 0.74, 95% CI: 0.64–0.85; *p* < 0.001, Figure 4A). The analysis was performed using a random-effects model (I^2^ = 57%). We then compared OS in patients whose PD-L1 expression was ≥1 and ≥5%. The pooled analysis in PD-L1 ≥ 1% cases showed improved OS in the experimental arm (HR = 0.72, 95% CI: 0.56–0.94; *p* = 0.02, Figure 4B). However, no better result was found in PD-L1 ≥ 5% cases (HR = 0.78, 95% CI: 0.68–0.90; *p* < 0.001, Figure 4B).

#### Objective Response Rate

**FIGURE 5:**
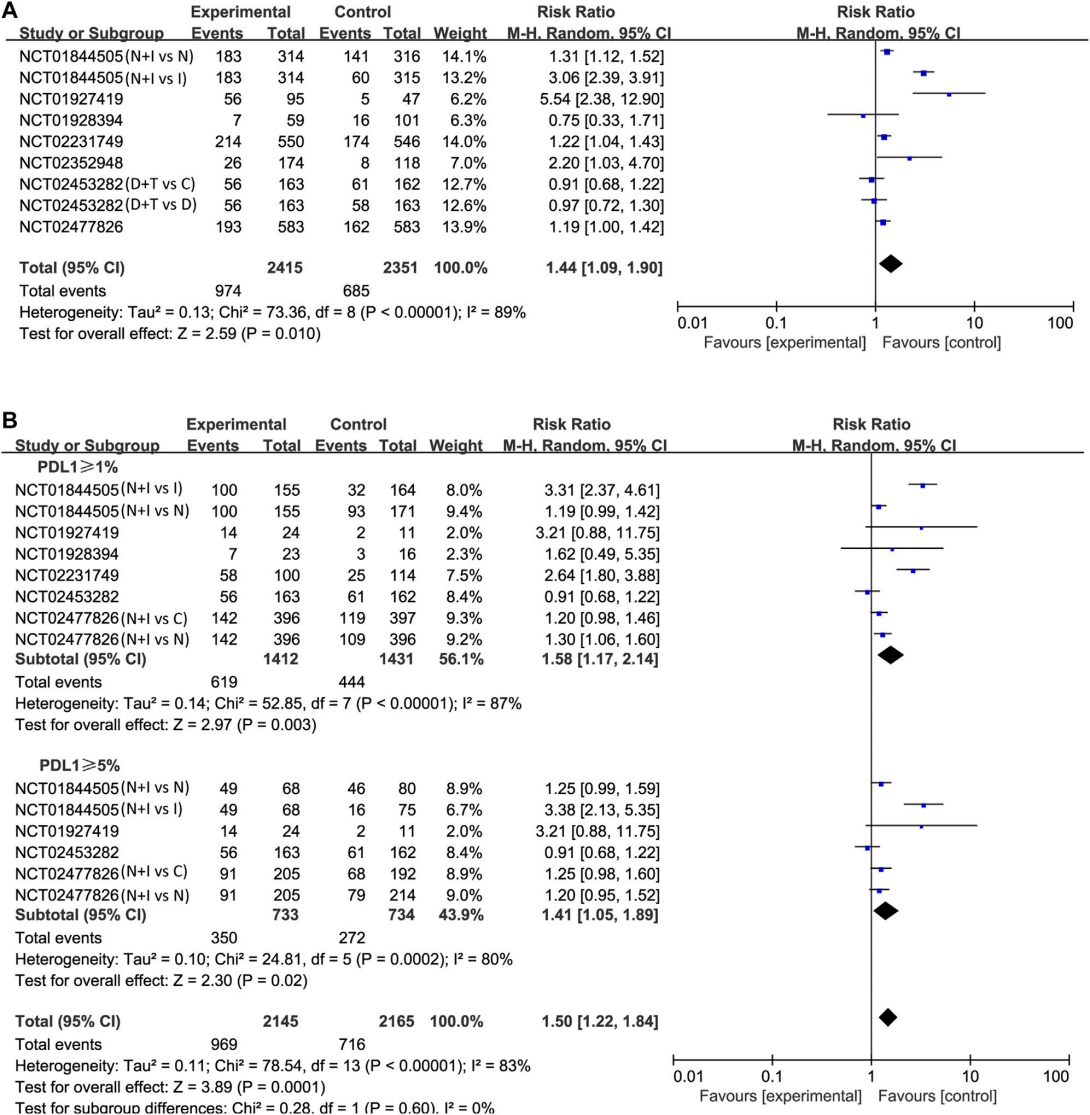
Forest plots of risk ratios (RRs) for objective response rate (ORR) comparing combination of immune checkpoint inhibitors with other treatments. **(A)** Unselected patients. **(B)** PD-L1 positive patients.

Lastly, the objective response was investigated in all studies. Using the Mantel–Haenszel method, the pooled RR was 1.44 (95% CI 1.09–1.90; *p* = 0.01; I^2^ = 89%, random effect model; Figure 5A) in treatment arm. This means that PD-1/PD-L1 combined with CTLA-4 has a higher objective response rate than other treatments. Also, we found similar results in patients with positive expression of PD-L1. In PD-L1 ≥ 1% cases, the pooled RR was 1.58 (95% CI 1.17–2.14; *p* = 0.003; I^2^ = 87%, random effect model; Figure 5B). In PD-L1 ≥ 5% cases, the pooled RR was 1.41 (95% CI 1.05–1.89; *p* = 0.02; I^2^ = 80%, random effect model; Figure 5B).

### Subgroup Analysis

In order to investigate sources of heterogeneity, subgroup analysis was undertaken based on different intervention measures. We mainly classify the intervention measures according to nivolumab plus ipilimumab *vs.* nivolumab and nivolumab plus ipilimumab vs ipilimumab (Supplementary Figures S1–S3). In PFS, when PD-L1 ≥ 1%, the pooled HR of nivolumab plus ipilimumab vs. nivolumab was 0.85 (95% CI, 0.74–0.97; *p* = 0.02), while the HR of nivolumab plus ipilimumab vs. ipilimumab was 0.39 (95% CI, 0.30–0.50; *p* < 0.001); PD-L1 ≥ 5%, the pooled HR were 0.82 (95% CI, 0.67–1.00; *p* = 0.05) and 0.34 (95% CI, 0.24–0.48; *p* < 0.001), respectively. The results suggested that intervention could be the potential sources of heterogeneity. Also, we found that using combination therapy was significantly better than the ipilimumab monotherapy. Similar results were found in OS and ORR. (Supplementary Figures S4–S6).

### Publication Bias Test and Sensitivity Analysis

Publication bias was not performed because no more than 10 studies were included. Sensitivity analysis was discussed based on the results. After switching the random effect model to the fixed effect model, the results did not change significantly, indicating that the results are relatively stable (Table 2).

**TABLE 2:** Sensitivity analysis.

	Total PFS	PFS (PD-L1 ≥ 1%)	PFS (PD-L1 ≥ 5%)	Total OS	OS (PD-L1 ≥ 1%)	OS (PD-L1 ≥ 5%)	Total ORR	ORR (PD-L1 ≥ 1%)	ORR (PD-L1 ≥ 5%)
Random effect model	0.69 (0.55–0.86)	0.61 (0.45–0.81)	0.57 (0.41–0.80)	0.74 (0.64–0.85)	0.72 (0.56–0.94)	0.78 (0.68–0.90)	1.44 (1.09–1.90)	1.58 (1.17–2.14)	1.41 (1.05–1.89)
Fixed effect model	0.71 (0.66–0.77)	0.72 (0.66–0.79)	0.64 (0.56–0.74)	0.73 (0.67–0.79)	0.78 (0.70–0.88)	0.78 (0.68–0.90)	1.39 (1.28–1.51)	1.42 (1.29–1.57)	1.30 (1.15–1.47)

PFS/OS: HR, 95%CI (Treatment group vs. control group); ORR: RR, 95%CI (Treatment group vs. control group).

## Discussion

This meta-analysis indicates that PD-1/PD-L1 combined with CTLA-4 has better therapeutic efficacy, compared with other treatments. Regardless of PD-L1 expression, the combination therapy shows longer PFS, OS and better ORR. In PFS, we found that the efficacy of combined immunotherapy was related to the expression of PD-L1, and the PFS of patients with PD-L1 ≥ 5% was longer than those with PD-L1 ≥ 1%. However, in OS and ORR, the survival benefit of cancer patients did not relate to the status of PD-L1. Therefore, we believed that the status of PD-L1 may not be a perfect biomarker in combination immunotherapy.

The presence of CTLA-4 can inhibit the co-stimulation of B7 and CD-28, thus inhibiting the proliferation of T cells (Lenschow et al., 1996). Similar to CTLA-4, PD-1 and its ligands play a role in down-regulating the immune system by blocking T cell activation, which in turn reduces autoimmune and promotes self-tolerance (Keir et al., 2006). PD-1 contributes to peripheral tissue T cells failure, while CTLA-4 inhibits T cells at an earlier stage of activation (Wolchok et al., 2013). They can suppress autoimmunity and promote immune tolerance by blocking the activation of T cells (Francisco et al., 2009). Hence, PD-1/PD-L1 and CTLA-4 play complementary roles in regulating adaptive immunity (Diesendruck and Benhar 2017). Clinically, several studies have shown that combination therapy has survival benefits in different types of tumors compared to other monotherapy (Wolchok et al., 2013; Robert et al., 2015; Antonia et al., 2016; Hellmann et al., 2017). And in October 2015, the FDA approved a melanoma regimen that combines anti-CTLA-4 (ipilimumab) with anti-PD1 (nivolumab) (Larkin et al., 2015). This result was also supported in our meta-analysis.

The status of PD-L1 as a biomarker to predict the efficacy of immune checkpoint inhibitors has always been controversial. KEYNOTE-001 in 2016 (Daud et al., 2016) and KEYNOTE-010 in 2014 (Herbst et al., 2014) showed that melanoma patients and non-small cell lung cancer patients with PD-L1 positive had a greater survival benefit from anti-PD-L1/anti-PD-1 therapy. A previous meta-analysis also showed that positive expression of PD-L1 in malignant tumors was significantly higher than negative expression in objective response rate (Gandini et al., 2016). In combination immunotherapy, however, different results were reported. CheckMate-067 in 2019 showed that the efficacy of nivolumab combine with ipilimumab in melanoma is not associated with the expression of PD-L1 (Larkin et al., 2015). Similar results were also shown in CheckMate-032 in 2018 (Janjigian et al., 2018). While Long et al. found that combination nivolumab and ipilimumab in melanoma brain metastases, patients with PD-L1 expression ≥1% had longer PFS than those with PD-L1 expression <1% (Long et al., 2018). Our study found that PFS was positively correlated with PD-L1 expression, while OS and ORR were not significantly correlated with PD-L1 expression. This may be related to tumor type or treatment line. So we performed subgroup analysis. The results revealed that the use of combined immunotherapy in the first-line treatment was superior to the third-line treatment, whether OS or PFS. However, the limited number of included trials prevented us to conduct further studies on the expression status of PD-L1. In terms of tumor types, we studied non-small cell lung cancer and malignant melanoma. The results suggested that the efficacy of combined immunotherapy in malignant melanoma was better than that in non-small-cell lung cancer, whether OS, PFS or ORR. Such results were also found in PD-L1 ≥ 1% and PD-L1 ≥ 5% cases, but their efficacy did not improve with the increase of PD-L1 expression (Supplementary Table S2). Due to the insufficient number of the eligible clinical trials, we were unable to evaluate other factors that may affect the results.

In our subgroup analysis, we found that the treatment of nivolumab combined with ipilimumab was obviously better than the treatment of ipilimumab monotherapy, while there was no significant advantage over nivolumab monotherapy in PD-L1 ≥1% and ≥5% cases. Since the dominant mechanism associated with anti-PD-1 drug response is PD-L1 expression (Topalian et al., 2016). Therefore, when PD-L1 is highly expressed, anti-PD-1 drugs are not suggested to use with anti-CTLA-4 drugs together, which reduces the toxic side effects and economic burden. In the two clinical trials included on durvalumab and tremelimumab combination therapy, we found almost no survival benefit or even negative effects (Planchard et al., 2020; Rizvi et al., 2020). However, the MYSTIC trial reflected that in patients with bTMB ≥ 20 mut/Mb, the OS and PFS of the durvalumab and tremelimumab combination therapy were considerably longer than those in the chemotherapy group (Rizvi et al., 2020). Therefore, tumor mutation burden might be one of the biomarkers of PD-1/PD-L1 combined with CTLA-4 treatment.

Indeed, combination immunotherapy has achieved promising results in terms of curative effect, but the adverse events should be considered. Previous meta-analyses showed that the incidence of fatal events in combination immunotherapy was higher than that in single immunotherapy, mainly respiratory diseases and cardiotoxicity, but less frequent. Gastrointestinal diseases, respiratory diseases and rashes were the most common grade 3–4 adverse reactions. Overall, the adverse effects of immunotherapy were manageable (Xu et al., 2019; Yang et al., 2020). Since adverse events have been discussed in previous studies (Xu et al., 2019; Yang et al., 2020), and will not be further considered here.

There were also several limitations should be observed. First, our study was based on literature research, resulting in some deviation of statistical results. Second, only seven clinical trials were included, and the control arm and the treatment arm are different. Third, the detection methods of PD-L1 are different in trials. PD-L1 itself has certain limitations, such as the tumor heterogeneity and the effect of PD-L1 expression on tumor cells and immune cells, etc.

## Conclusion

Combination immunotherapy has become the focus of discussion in recent years, and many related clinical studies have been reported. However, research on biomarkers related to combined immunotherapy remains controversial. Our meta-analysis revealed that PFS in patients with malignant tumors is positively correlated with PD-L1 expression, since the conclusions were drawn from a small number of clinical trials. More large-sample, multicenter and well-designed randomized controlled trials are still expected.

## Data Availability

The original contributions presented in the study are included in the article/Supplementary Material, further inquiries can be directed to the corresponding authors.
