# Supplementary figures and images for: Results from a Meta-analysis of Combination of PD-1/PD-L1 and CTLA-4 Inhibitors in Malignant Cancer Patients: Does PD-L1 Matter?
Source: Front Pharmacol. 2021 Feb 25;12:572845. doi: 10.3389/fphar.2021.572845 (PMC7949479; doi:10.3389/fphar.2021.572845)

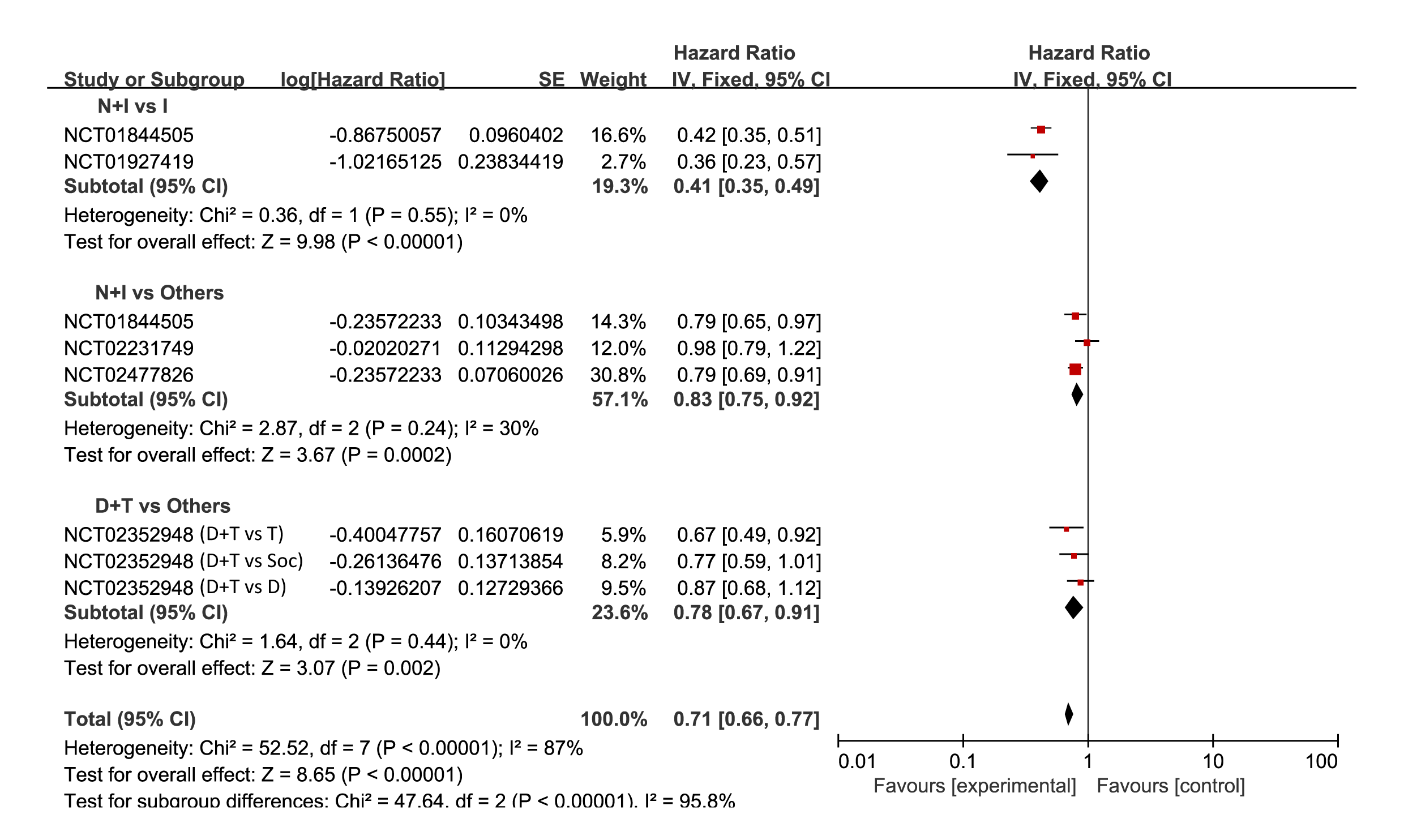

Supplement: Supplementary file 1 [file image1.tif]

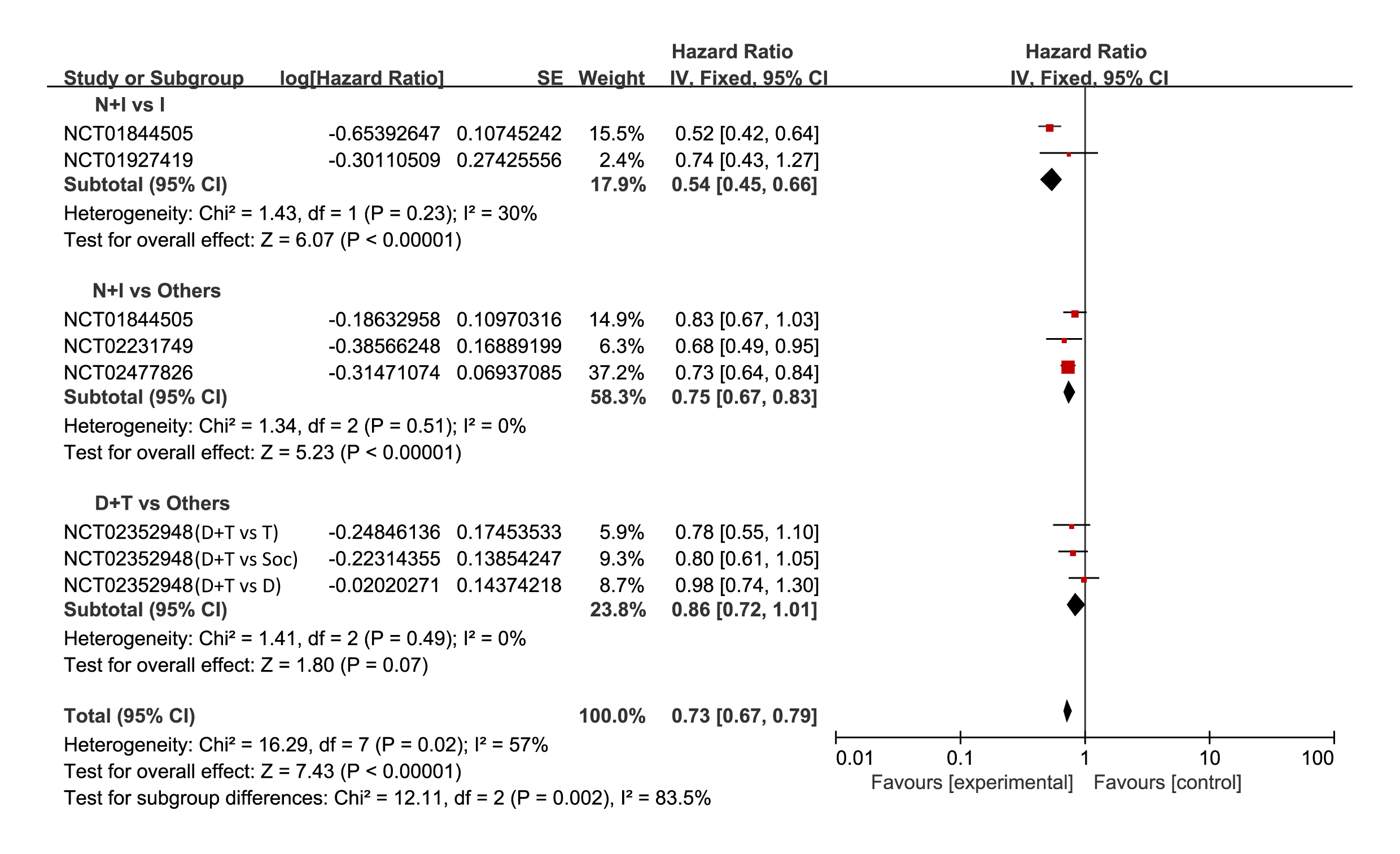

Supplement: Supplementary file 2 [file image2.tif]

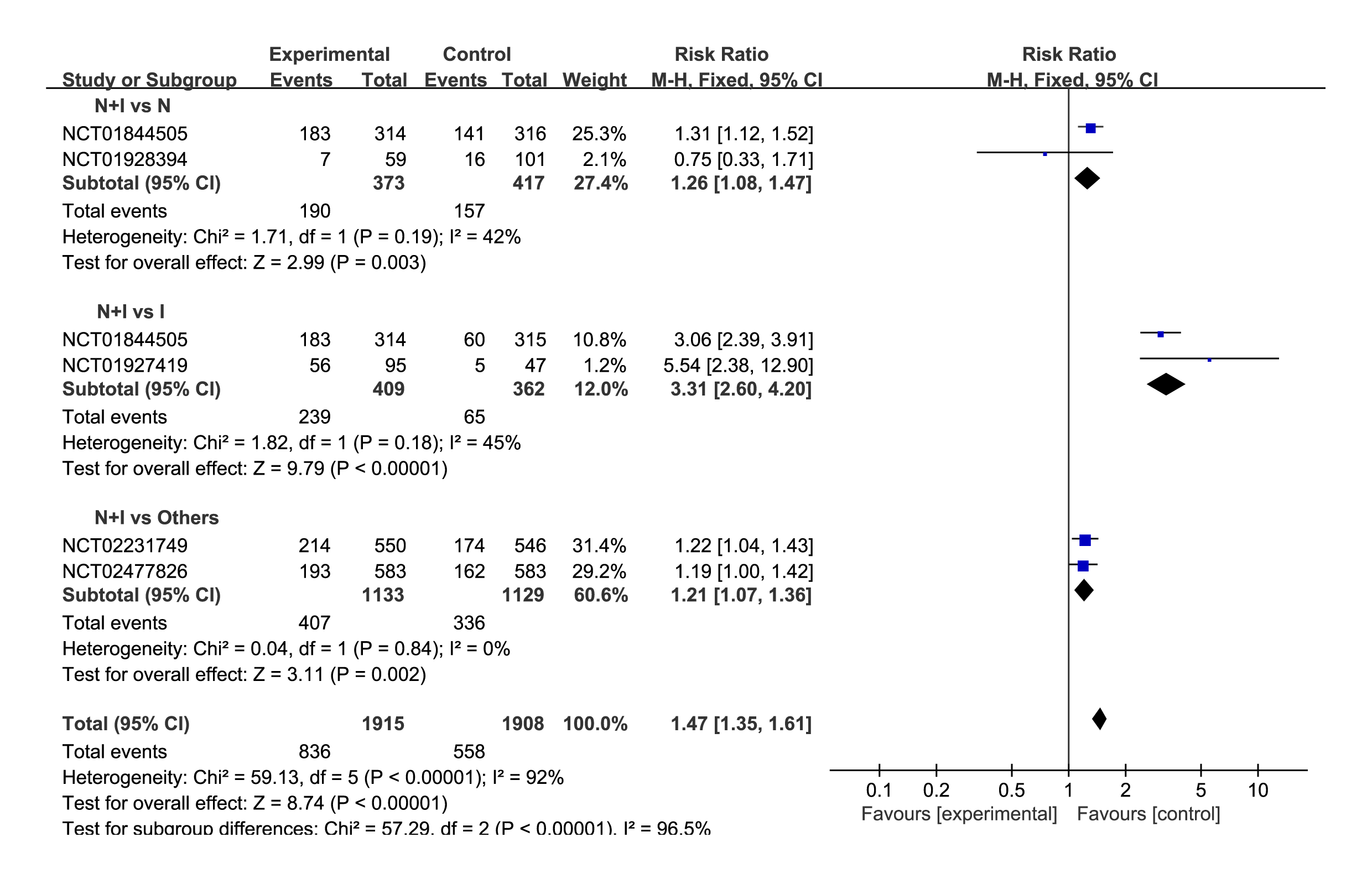

Supplement: Supplementary file 3 [file image3.tif]

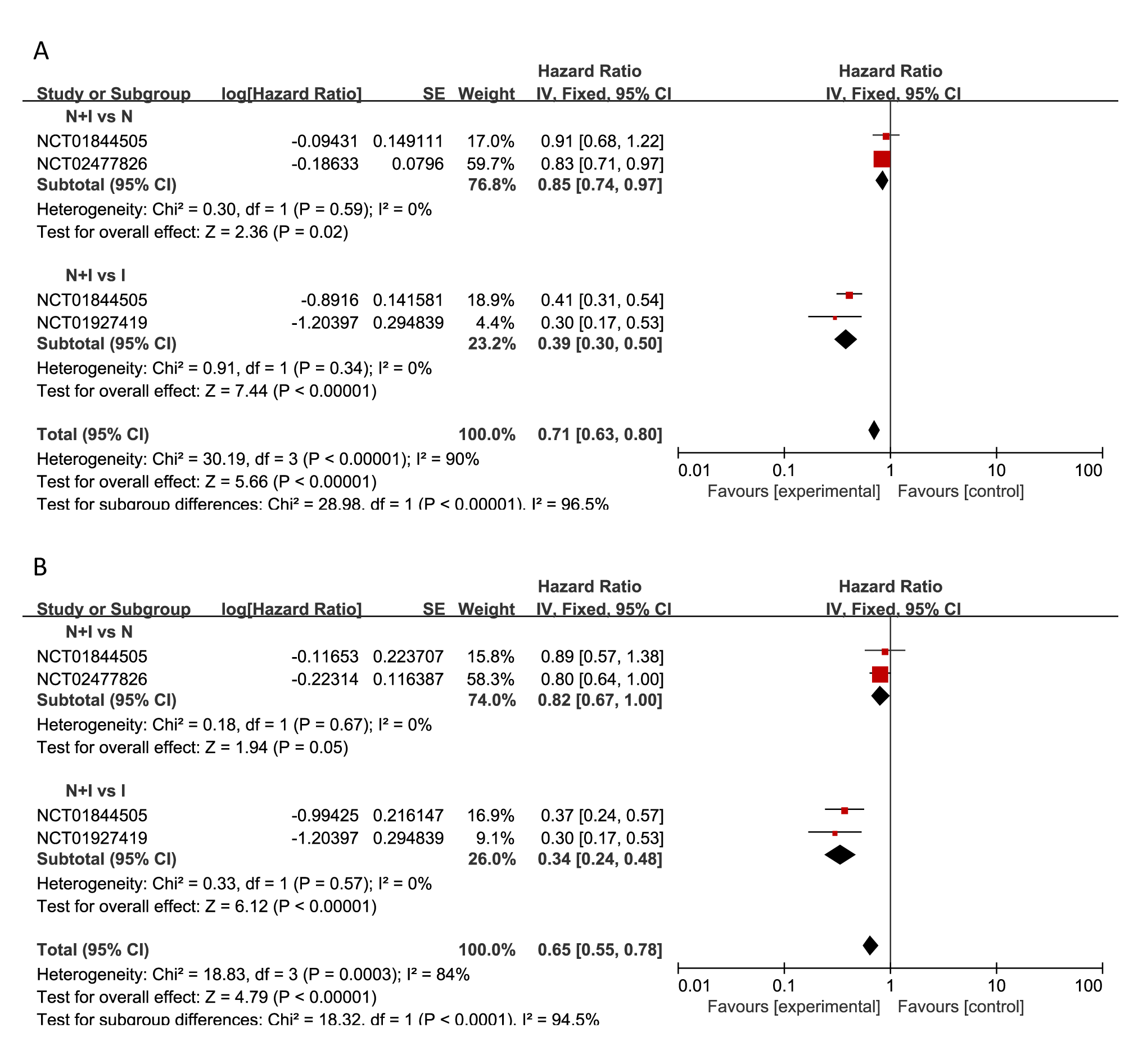

Supplement: Supplementary file 4 [file image4.tif]

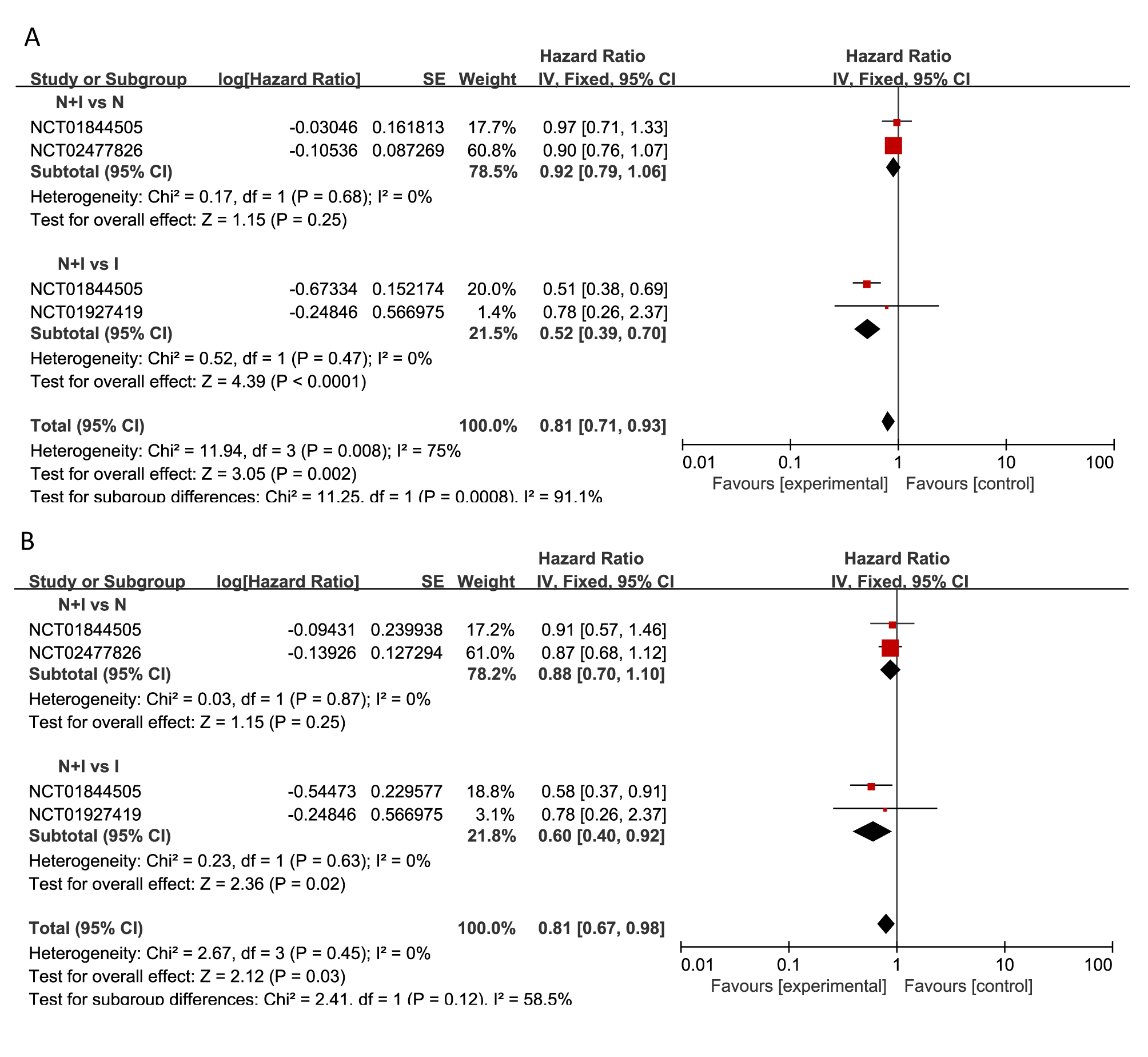

Supplement: Supplementary file 5 [file image5.tif]
