# Supplementary material for: Results from a Meta-analysis of Combination of PD-1/PD-L1 and CTLA-4 Inhibitors in Malignant Cancer Patients: Does PD-L1 Matter?
Source: Front Pharmacol. 2021 Feb 25;12:572845. doi: 10.3389/fphar.2021.572845 (PMC7949479; doi:10.3389/fphar.2021.572845)
Supplement: Supplementary file 6 [file table1.docx]

| Study | PFS  (months,95% CI) |  | OS  (months,95% CI) |  | Objective response rate (%) |  |
| --- | --- | --- | --- | --- | --- | --- |
|  | Exp arm | C arm | Exp arm | C arm | Exp arm | C arm |
| NCT01844505 | 11.5(8.7-19.3) | 6.9(5.1-10.2)/  2.9(2.8-3.2) | NR(38.2-NR) | 36.9(28.2-58.7)/  19.9(16.8-24.6) | 58 | 45/  19 |
| NCT01927419 | NR | 3(2.7-5.1) | NA | NA | 59 | 11 |
| NCT01928394 | 1.4(1.2-3.8) | 1.4(1.2-1.5) | 6.9(3.7-11.5) | 6.2(3.4-12.4) | 12 | 16 |
| NCT02231749 | 12.4(9.9-16.5) | 12.3(9.8-15.2) | NR | 32.9(NA) | 39 | 32 |
| NCT02352948 | 3.5(2.3-4.6) | 3.5(1.9-3.9)/  3.1(1.9-3.7)/  2.1(1.8-3.2) | 11.5(8.7-14.1) | 8.7(6.5-11.7)/  10.0(7.1-13.2)/  6.9(3..9-13.2) | 15 | 7 |
| NCT02453282 | 3.9(2.8-5.0) | 5.4(4.6-5.8)/  NA | 11.9(9.0-17.7) | 12.9(10.5-15)/  16.3(12.2-20.8) | 36 | 34/  38 |
| NCT02477826 | NA | NA | 17.1(15.2-19.9) | 13.9(12.2-15.1) | 33 | 28 |
| PDL1≥1% | | | | | | |
| NCT01844505 | 16.1(8.9-22.2) | 16.2(8.1-27.7)/  3.5(2.8-4.2) | NR(39.1-NR) | 67.0(39.0-NR)/  21.5(16.9-29.1) | 65 | 54/  20 |
| NCT01928394 | NA | NA | NA | NA | 30 | 19 |
| NCT02231749 | 22.8(9.4-NR) | 5.9(4.4-7.1) | NR | 19.6(14.8-NE) | 58 | 22 |
| NCT02477826 | NA | NA | 17.1(15.0-22.1) | 14.9(12.7-16.7)(C) | 35.9 | 27.5/  30.0 |
| PDL1≥5% | | | | | | |
| NCT01844505 | 22.1(9.7-NR) | 22.3(9.5-40.0)/  3.9(2.8-4.2) | NR(39.1-NR) | 61.6(33.6-NR)/  28.9(18.1-44.2) | 72 | 58/  21 |
| NCT01927419 | 9.9(4.1-NR) | 3.0(2.1-NR) | NR(9.9-NR) | NR(4.8-NR) | 58 | 18 |
| PDL1≥10% | | | | | | |
| NCT01844505 | 47.1(14.0-NR) | 22.0(9.1-39.1)/  4.1(2.8-5.6) | NR(39.1-NR) | 43.6(31.2-NR)/  29.1(17.4-46.6) | 85 | 58/  20 |
| PDL1≥25% | | | | | | |
| NCT02453282 | 3.9(2.8-5.0) | 5.4(4.6-5.8)/  NA | 11.9(9.0-17.7) | 12.9(10.5-15)/  16.3(12.2-20.8) | 36 | 34/  38 |
| PDL1≥50% | | | | | | |
| NCT02477826 | NA | NA | NA | NA | 44 | 37/  35 |

NA: Not available; NR: Not reached; NE: Not estimable.
