# Supplementary material for: Results from a Meta-analysis of Combination of PD-1/PD-L1 and CTLA-4 Inhibitors in Malignant Cancer Patients: Does PD-L1 Matter?
Source: Front Pharmacol. 2021 Feb 25;12:572845. doi: 10.3389/fphar.2021.572845 (PMC7949479; doi:10.3389/fphar.2021.572845)
Supplement: Supplementary file 7 [file table2.docx]

|  | **Total PFS** | **PFS (PD-L1≥1%)** | **PFS (PD-L1≥5%)** | **Total OS** | **OS (PD-L1≥1%)** | **OS (PD-L1≥5%)** | **Total ORR** | **ORR (PD-L1≥1%)** | **ORR (PD-L1≥5%)** |
| --- | --- | --- | --- | --- | --- | --- | --- | --- | --- |
| **Treatment Line** | | | | | | | | | |
| First Line | 0.69  (0.63-0.76) | 0.72  (0.66-0.79) | 0.64  (0.56-0.74) | 0.69  (0.63-0.76) | 0.72  (0.56-0.94) | 0.78  (0.68-0.90) | 1.39  (1.28-1.51) | 1.42  (1.29-1.57) | 1.30  (1.15-1.47) |
| ≥Third Line | 0.78  (0.67-0.91) | ------- | ------- | 0.86  (0.72-1.01) | ------ | ------ | 1.40  (0.81-2.40) | ------ | ------ |
| **Cancer Type** | | | | | | | | | |
| Melanoma | 0.54  (0.48-0.62) | 0.56  (0.46-0.67) | 0.49  (0.38-0.64) | 0.66  (0.57-0.76) | 0.71  (0.42-1.20) | 0.72  (0.53-0.99) | 1.95  (1.71-2.23) | 1.77  (1.51-2.08) | 1.88  (1.51-2.34) |
| Non–Small-Cell Lung Cancer | 0.79  (0.71-0.87) | 0.83  (0.74-0.93) | 0.71  (0.60-0.84) | 0.78  (0.70-0.86) | 0.89  (0.76-1.03) | 0.79  (0.68-0.93) | 1.12  (0.98-1.28) | 1.18  (1.03-1.34) | 1.13  (0.98-1.31) |

PFS/OS: HR, 95%CI ( Treatment group vs control group); ORR: RR, 95%CI (Treatment group vs control group)
