# Supplementary material for: Results from a Meta-analysis of Combination of PD-1/PD-L1 and CTLA-4 Inhibitors in Malignant Cancer Patients: Does PD-L1 Matter?
Source: Front Pharmacol. 2021 Feb 25;12:572845. doi: 10.3389/fphar.2021.572845 (PMC7949479; doi:10.3389/fphar.2021.572845)
Supplement: Supplementary file 8 [file table3.docx]

PUBMED 635

(((((randomized controlled trial[Publication Type] OR controlled clinical trial[Publication Type] OR randomized[Title/Abstract] OR placebo[Title/Abstract] OR randomly[Title/Abstract] OR trial[Title/Abstract] OR groups[Title/Abstract]))) NOT ((animals[MeSH Terms]) NOT humans[MeSH Terms]))) AND ((((CTLA-4 Antigen[MeSH Terms] OR CTLA-4[Title/Abstract] OR CTLA4[Title/Abstract] OR CD152[Title/Abstract] OR Cytotoxic TLymphocyteAssociated Antigen 4[Title/Abstract] OR Cytotoxic T-Lymphocyte Antigen 4[Title/Abstract] OR Ipilimumab[Title/Abstract] OR Tremelimumab[Title/Abstract]))) AND ((((((((((((((((((((((((((Programmed Cell Death 1 Receptor[MeSH Terms]) OR PD-1 Receptor[Title/Abstract]) OR PD1 Receptor[Title/Abstract]) OR Receptor, PD1[Title/Abstract]) OR Programmed Cell Death 1 Protein[Title/Abstract]) OR Programmed death receptor 1[Title/Abstract]) OR PD-1[Title/Abstract]) OR Programmed death ligand1[Title/Abstract]) OR PD-L1[Title/Abstract]) OR Programmed Cell Death 1 Receptor[Title/Abstract]) OR nivolumab[Title/Abstract]) OR pembrolizumab[Title/Abstract]) OR atezolizumab[Title/Abstract]) OR durvalumab[Title/Abstract]) OR cemiplimab[Title/Abstract]) OR toripalimab[Title/Abstract]) OR sintilimab[Title/Abstract]) OR avelumab[Title/Abstract]) OR Keytruda[Title/Abstract]) OR Opdivo[Title/Abstract]) OR Libtayo[Title/Abstract]) OR Tecentriq[Title/Abstract]) OR Bavencio[Title/Abstract]) OR Imfinzi[Title/Abstract]) OR Camrelizumab[Title/Abstract])))

EMBASE 1566

#48 #12 AND #42 AND #47 1,566

#47 #43 OR #44 OR #45 OR #46 871,110

#46 'double blind procedure'/exp 170,765

#45 'randomization'/exp 86,178

#44 'randomized controlled trial (topic)'/exp 176,006

#43 'randomized controlled trial'/exp 596,370

#42 #13 OR #14 OR #15 OR #16 OR #17 OR #18 OR #19 OR #20 OR #21 OR #22 OR #23 OR #24 OR #25 OR #26 OR #27 OR #28 OR #29 OR #30 OR #31 OR #32 OR #33 OR #34 OR #35 OR #36 OR #37 OR #38 OR #39 OR #40 OR #41 56,895

#41 'camrelizumab':ab,ti 35

#40 'camrelizumab'/exp 64

#39 'avelumab':ab,ti 831

#38 'avelumab'/exp 2,044

#37 'sintilimab':ab,ti 31

#36 'sintilimab'/exp 49

#35 'toripalimab':ab,ti 18

#34 'toripalimab'/exp 55

#33 'cemiplimab':ab,ti 119

#32 'cemiplimab'/exp 237

#31 'durvalumab':ab,ti 1,296

#30 'durvalumab'/exp 3,346

#29 'atezolizumab':ab,ti 2,093

#28 'atezolizumab'/exp 4,836

#27 'pembrolizumab':ab,ti 7,820

#26 'pembrolizumab'/exp 13,948

#25 'nivolumab':ab,ti 9,730

#24 'nivolumab'/exp 16,197

#23 'programmed cell death 1 receptor':ab,ti 98

#22 'pd-l1':ab,ti 23,117

#21 'pd-1':ab,ti 27,873

#20 'programmed death receptor 1':ab,ti 500

#19 'programmed cell death 1 protein':ab,ti 42

#18 'receptor, pd1':ab,ti 53

#17 'pd1 receptor':ab,ti 63

#16 'pd-1 receptor':ab,ti 781

#15 'programmed death ligand1':ab,ti 21

#14 'programmed death 1 receptor':ab,ti 221

#13 'programmed death 1 receptor'/exp 20,416

#12 #1 OR #2 OR #3 OR #4 OR #5 OR #6 OR #7 OR #8 OR #9 OR #10 OR #11 33,626

#11 'cytotoxic t-lymphocyte antigen 4':ab,ti 1,963

#10 'cytotoxic tlymphocyteassociated antigen 4':ab,ti 1

#9 'ctla4':ab,ti 5,790

#8 'ctla-4':ab,ti 11,408

#7 'ctla-4 antigen':ab,ti 13

#6 'cytotoxic t lymphocyte antigen 4'/exp 19,630

#5 'ticilimumab':ab,ti 10

#4 'ticilimumab'/exp 2,175

#3 'tremelimumab':ab,ti 602

#2 'ipilimumab':ab,ti 6,461

#1 'ipilimumab'/exp 13,239

COCHRANE 431

ID Search Hits

#1 MeSH descriptor: [CTLA-4 Antigen] explode all trees 39

#2 (Cytotoxic T Lymphocyte Associated Antigen 4):ti,ab,kw (Word variations have been searched) 192

#3 (CD152 Antigen):ti,ab,kw (Word variations have been searched) 10

#4 (Antigen, CD152):ti,ab,kw (Word variations have been searched) 10

#5 (Cytotoxic T Lymphocyte Antigen 4):ti,ab,kw (Word variations have been searched) 398

#6 (CTLA 4 Antigen):ti,ab,kw (Word variations have been searched) 196

#7 (Cytotoxic T-Lymphocyte-Associated Antigen 4):ti,ab,kw (Word variations have been searched) 64

#8 (Antigen, CTLA-4):ti,ab,kw (Word variations have been searched) 193

#9 (Antigens, CD152):ti,ab,kw (Word variations have been searched) 10

#10 (Cytotoxic T-Lymphocyte Antigen 4):ti,ab,kw (Word variations have been searched) 381

#11 MeSH descriptor: [Ipilimumab] explode all trees 140

#12 (Ipilimumab):ti,ab,kw 1035

#13 (Yervoy):ti,ab,kw (Word variations have been searched) 56

#14 (ticilimumab):ti,ab,kw (Word variations have been searched) 43

#15 (tremelimumab):ti,ab,kw (Word variations have been searched) 229

#16 #1 OR #2 OR #3 OR #4 OR #5 OR #6 OR #7 OR #8 OR #9 OR #10 OR #11 OR #13 OR #14 OR #15 788

#17 MeSH descriptor: [Programmed Cell Death 1 Receptor] explode all trees 45

#18 (Programmed Cell Death 1 Receptor):ti,ab,kw (Word variations have been searched) 436

#19 (Programmed Cell Death 1 Protein):ti,ab,kw (Word variations have been searched) 338

#20 (PD 1 Receptor):ti,ab,kw (Word variations have been searched) 3499

#21 (Receptor, PD-1):ti,ab,kw (Word variations have been searched) 490

#22 (Receptor, PD1):ti,ab,kw (Word variations have been searched) 54

#23 (Antigen, CD279):ti,ab,kw (Word variations have been searched) 4

#24 (programmed death receptor 1):ti,ab,kw (Word variations have been searched) 568

#25 (pd-1):ti,ab,kw (Word variations have been searched) 1558

#26 (pd-l1):ti,ab,kw (Word variations have been searched) 1822

#27 MeSH descriptor: [Nivolumab] explode all trees 372

#28 (nivolumab):ti,ab,kw (Word variations have been searched) 1498

#29 (pembrolizumab):ti,ab,kw (Word variations have been searched) 1292

#30 (atezolizumab):ti,ab,kw (Word variations have been searched) 620

#31 (durvalumab):ti,ab,kw (Word variations have been searched) 456

#32 (cemiplimab):ti,ab,kw (Word variations have been searched) 32

#33 (toripalimab):ti,ab,kw (Word variations have been searched) 16

#34 (sintilimab):ti,ab,kw (Word variations have been searched) 26

#35 (avelumab):ti,ab,kw (Word variations have been searched) 170

#36 (Keytruda):ti,ab,kw (Word variations have been searched) 69

#37 (Opdivo):ti,ab,kw (Word variations have been searched) 85

#38 (Tecentriq):ti,ab,kw (Word variations have been searched) 50

#39 (Bavencio):ti,ab,kw (Word variations have been searched) 5

#40 (Imfinzi):ti,ab,kw (Word variations have been searched) 5

#41 (Camrelizumab):ti,ab,kw (Word variations have been searched) 16

#42 #17 OR #18 OR #19 OR #20 OR #21 OR #22 OR #23 OR #24 OR #25 OR #26 OR #27 OR #28 OR #29 OR #30 OR #31 OR #32 OR #33 OR #34 OR #35 OR #36 OR #37 OR #38 OR #39 OR #40 OR #41 7501

#43 #42 AND #16 432
